# Evaluation of spore inoculum and confirmation of pathway genetic blueprint of T13αH and DBAT from a Taxol-producing endophytic fungus

**DOI:** 10.1038/s41598-020-77605-x

**Published:** 2020-12-03

**Authors:** Balabhadrapatruni V. S. K. Chakravarthi, Satpal Singh, Subban Kamalraj, Vijai Kumar Gupta, Chelliah Jayabaskaran

**Affiliations:** 1 Department of Biochemistry, Indian Institute of Science, Bangalore, 560012 India; 2 Biorefining and Advanced Materials Research Center, Scotland’s Rural College (SRUC), SRUC Barony Campus, Parkgate, Dumfries, DG1 3NE UK

**Keywords:** Biotechnology, Chemical biology, Microbiology, Molecular biology, Plant sciences

## Abstract

Taxol (paclitaxel), a plant-derived anticancer drug, has been among the most successful anticancer drugs of natural origin. Endophytic fungi have been proposed as a prominent alternative source for Taxol and its intermediate Baccatin III, however the very low yields remain a hinderance to their commercial utilization. Significant research efforts towards this end are underway globally. Here, we report the results on our earlier reported Taxol-producing endophytic fungus, *Fusarium solani* from the standpoint of spores as seed inoculum and media selection for enhanced Taxol and baccatin III yields. Spores produced on M1D medium with 94.76% viability were used for further media optimization for Taxol and Baccatin III production in five different liquid media under static and shaker condition at different cultivation days. Taxol and Baccatin III when quantified through competitive inhibition enzyme immunoassay (CIEIA), showed maximum production at 136.3 µg L^−1^ and 128.3 µg L^−1^, respectively in the modified flask basal broth (MFBB) under shaking condition. Further, two important genes of this pathway, namely taxane 13α-hydroxylase (T13αH) and 10-deacetylbaccatin III-10-β-O-acetyltransferase (DBAT) have been identified in this fungus. These findings are hoped to assist in further manipulation and metabolic engineering of the parent *F. solani* strain towards the enhanced production of Taxol and baccatin III.

## Introduction

The diterpenoid taxane, Taxol (generic name Paclitaxel), remains a blockbuster anticancer drug having been used for decades either alone or in combination with other anticancer drugs for a variety of cancers including the AIDS-related Kaposi sarcoma^1^. Upon its discovery in early sixties from the first reported source, the Pacific yew tree—*Taxus brevifolia*^2^, mainly from its bark as the main tissue of production, the subsequent decades saw the ecological concerns in destruction of a large number of trees putting a roadblock in its supply to the increasing worldwide demand. This eventually resulted in advancing research on its alternate sources among which the endophytic fungi too appeared as a viable option since the first discovery of one such fungus from *Taxus brevifolia*^3,4^. This was followed by several other unrelated Taxol-producing endophytic fungi from genera such as *Taxomyces, Pestalotiopsis, Fusarium,* etc^5^. Among the many fungi reported so far to produce Taxol, most have shown low Taxol yields from low to high micrograms per liter of culture. Microbial production is not only an inexpensive, streamlined method of production- but also offers the possibilities of finding new analogues with differential drug potential. The biosynthesis of Taxol from plant primary metabolism is a very complex process, including at least 20 enzymatic steps to construct its tetracyclic skeleton and the addition of the various hydroxyl and acyl functional groups^6^. Over the past couple of decades, several genes from the Taxol biosynthesis in *Taxus* spp. have been cloned and characterized such as geranylgeranyldiphosphate synthase, taxa-4(5),11(12)-diene synthase^7^, taxa-4(5),11(12)-diene-5α-hydroxylase, taxa-4(5),11(12)-diene-5α-ol-O-acetyltranseferase, taxane-10β-hydroxylase^7^, taxane 13α-hydroxylase^6^, taxoid 14-hydroxylase^8^, taxane 2α-O-benzoyltransferase, debenzoyltaxane-2′-α-O-benzoyltransferase, 10-deacetylbaccatin III-O-acetyltransferase, baccatin III 13-O-(3-amino-3-phenylpropanoyl) transferase (BAPT), 3′-N-debenzoyl-2′-deoxytaxol-N-benzoyltransferase^9^, (DBTNBT), phenylalanineaminomutase (PAM) and β-phenylalanine coenzyme A ligase^10^, etc.; however, several of the pathway genes still need to be cloned and characterized which is one of the main problems in engineered production of Taxol in *Taxus* host plants as well microbial sources. Some of the Taxol biosynthetic genes such as taxa-4(5),11(12)-diene synthase (TS), taxa-4(5),11(12)-diene-5α-ol-O-acetyltranseferase (TAT), taxane-10β-hydroxylase (T10βH), 10-deacetylbaccatin III-O-acetyltransferase (DBAT) and baccatin III 13-O-(3-amino-3-phenylpropanoyl) transferase (BAPT) genes have been reported in fungi^11^. It is quite difficult to find and clone these genes from fungi^12^ which leaves the hope to keep up the efforts in this direction vis-à-vis Taxol producing endophytic fungi. Previously, our laboratory had reported the isolation of an endophytic fungus from the stem cuttings of *Taxus celebica* which produced paclitaxel, 10-deacetylbaccatin III and baccatin III in liquid-grown cultures^13^. Besides paclitaxel, the fungus also yielded baccatin III and 10-deacetylbaccatin III which are important intermediates for paclitaxel and semisynthesis of paclitaxel in industry^14^. Fungal Taxol and baccatin III were characterized for their cytotoxic activity towards Jurkat cells in vitro^15^. The amount of fungal Taxol produced by *F. solani* was, however, relatively low at 1.6 μg L^−1^. Therefore, the current study was envisaged to find suitable media for Taxol/taxane production by *F. solani* using spores as the primary inoculum. For an industrially important fungus such as *F. solani*^13,16^, need for characterization of growth and sporulation with reference to Taxol production is an important requirement and desired goal. This is important in the context of mycelial growth, spore production and spore germination which began to be investigated more intensively for fungi parallel to the studies on media optimization^17,18^ thus highlighting the growing role of fungi in medicine, food industry and agriculture. Further, the action of light on the process of growth and spore formation followed by metabolic changes in the mycelia grown from these spores and enhancement of sporogenesis had been previously reported^19^. Interestingly, endophytic fungus SSM001 required the dark condition for the production of Taxol^20^ but the fungus was cultured using 1-week old fungal tips as inocula. The effects on the fungal spore seeding offers a possibility to increase the yield of the natural product in production media^21^. Most importantly, the morphological changes such as spore dormancy, spore swelling, germ tube formation and germ tube elongation during spore germination in liquid media affect the final secondary metabolites production potential^19^. Based on this background and in continuation of our investigations on the optimization of culture condition by improving culture growth and sporulation ultimately affecting the Taxol and baccatin III production potential of *F. solani*, we undertook the present studies on using spores as seed inoculum in addition to the media optimization under static and shake flask conditions as a function of cultivation time. In addition, we report the cloning of two Taxol biosynthetic genes—taxane-13α-hydroxylase (T13αH) and 10-deacetylbaccatin III-10-β-O-acetyltransferase (DBAT) from *F. solani* confirming the independent genetic blueprints of Taxol biosynthetic pathway in this fungus.

## Results

### *F*.* solani* shows media- and temperature-dependent radial mycelial growth differentially affected by the light regime applied

The radial growth of *F*. *solani* when tested on five different solid media (PDA, M1DA, S7A, FBA and MFBA) and five different temperatures (20, 25, 30, 35 and 40 °C) showed the maximum and minimum growths of 89 mm (at 30 °C) and 4.9 mm (at 40 °C) on MFBA and PDA, respectively after 12 days of incubation (Supplementary Fig. S1). The radial growth observed at 20 °C, 25 °C and 35 °C on all the five media showed moderate growth. The three different light regimes tested on five media too displayed a differential effect on the radial growth of *F*. *solani*. The maximum radial mycelial growth was observed at 87 mm under 12 h light/12 h dark cycles on MFBA medium followed by continuous 24 h light (82 mm) on the same medium. The growth was however, found to be significantly slower under the continuous 24 h dark regime on M1DA, FBA and MFBA (Supplementary Fig. S2). On the other hand, the growth was apparently unaffected by the light regime on PDA and S7A media (Supplementary Fig. S2). These data suggest the MFBA is the best medium to support optimal growth of the fungus.

### *F. solani* displays differential sporulation and spore germination in different media

**Figure 1:**
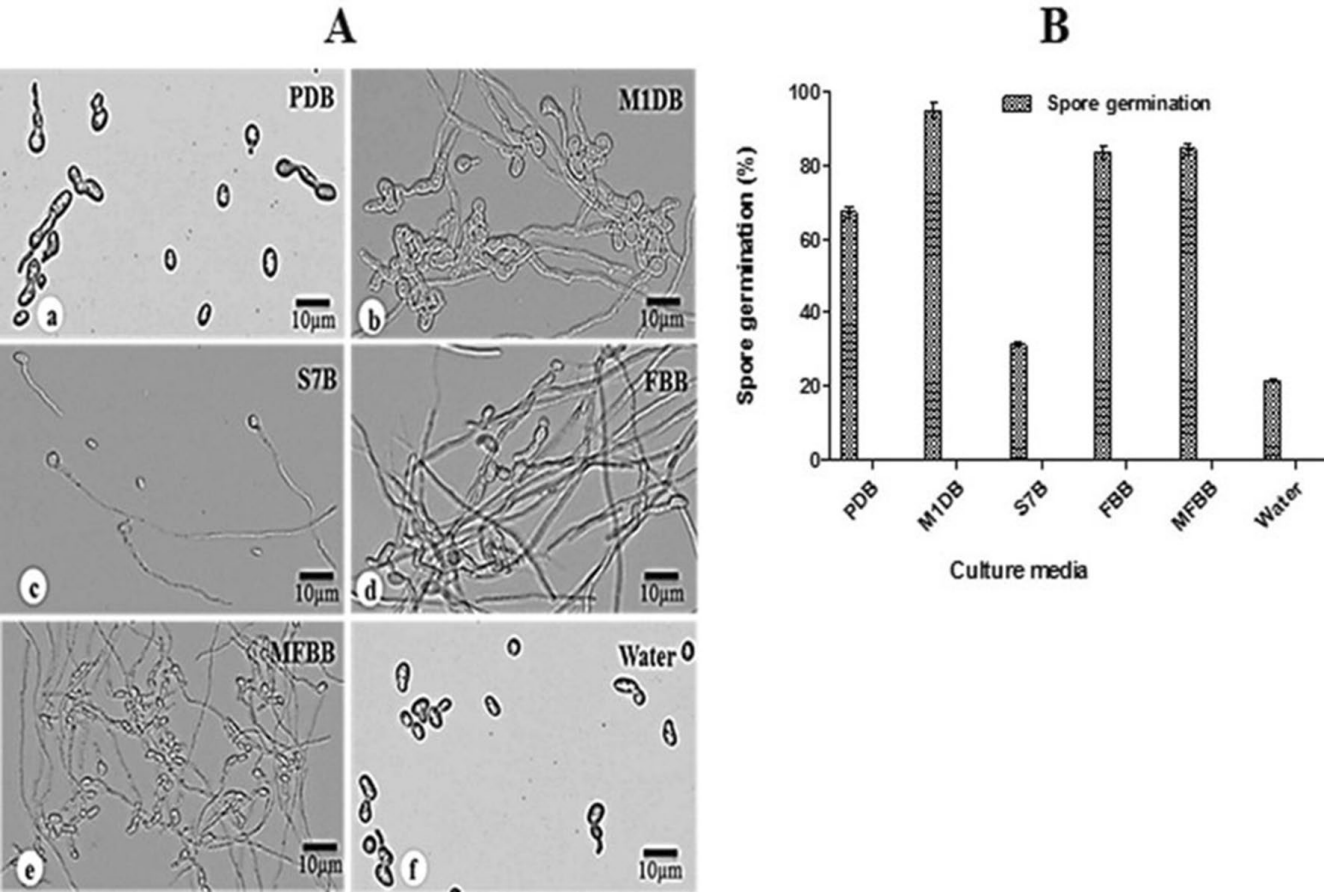
Microscopic visualization (**A**) and quantitative representation of the *F. solani* spore germination in five different liquid media. The fungus was cultured, spores harvested and tested for germination as described in “Materials and methods” section. Bar diagram in (**B**) shows the spore germination percentage expressed as means ± SD from three independent experiments. *PDB* potato dextrose broth, *M1DB* modified 1D broth, *S7B* antibiotic production broth, *FBB* flask basal broth, *MFBB* modified flask basal broth.

Our results showed that maximum sporulation was observed in PDA followed by M1DA medium. The descending order of spore production in units of spores mL^−1^ is 6.64 × 10^5^, 5.15 × 10^5^ mL^−1^, 1.94 × 10^5^ mL^−1^, 1.17 × 10^5^ mL^−1^ and 0.88 × 10^5^ mL^−1^ for PDA, M1DA, MFBA, FBA and S7A, respectively (Supplementary Fig. S3). Spores obtained from the PDA plates being highest in yield across all the five media tested were then evaluated for their germination in the same five media in liquid form (PDB, M1DB, S7B, FBB and MFBB). The results showed the highest germination of these spores in M1DB medium implying that the metabolic activity increases to support the germ tube emergence and elongation. Microscopic results showed significant germ-tube emergence followed by mycelial formation in M1DB, MFBB and FBB media while the same was observed to be only moderate and considerably delayed in S7B and PDB, respectively. In PDB only the spore swelling was visible at earlier time points (Fig. 1A). The maximum spore germination was obtained in M1DB (94.76%) followed by MFBB (84.27%) and FBB (83.51%). This could be attributed to the presence of certain media components, acting individually or in combination as stimulating factors in these defined media. The least conidial germination was recorded in the control sample of sterile distilled water (21.50%). The spore germination seems to be affected by the complexity of sugars either in form of simple or complex sugars as the maximum germination has been shown by the media having simple sugars while the germination in PDB having complex sugars was much lower at 67.23% (Fig. 1B).

### Five production media tested show discrete effect on the *F. solani* biomass in the liquid grown cultures

**Figure 2:**
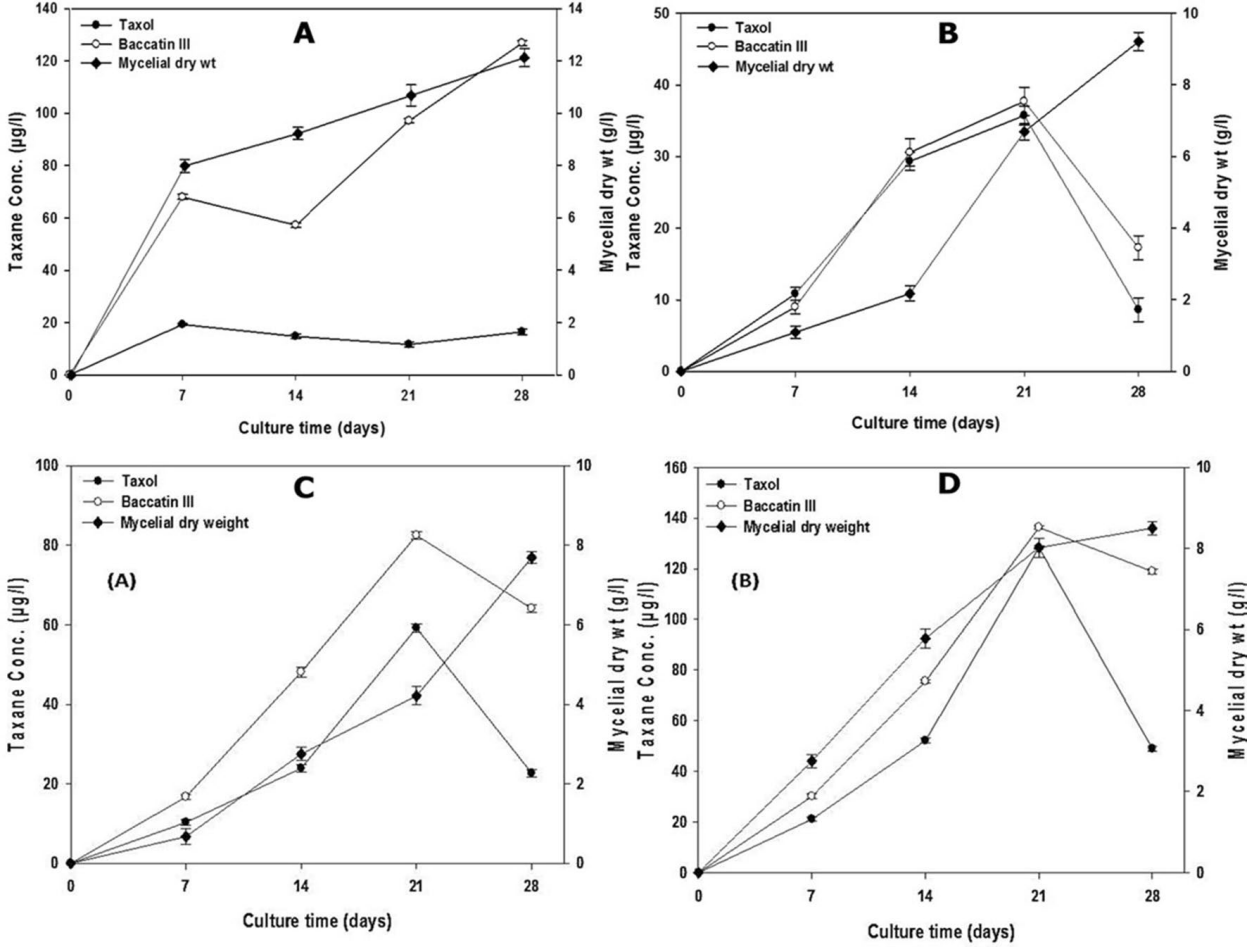
Time course of growth, Taxol and baccatin III production by *F. solani* in FBB and MFBB under static and shaking conditions. Panels **A** and **B** show the growth and production in FBB under static and shaking conditions, respectively while the production in MFBB under static and shaking conditions are shown in panels **C** and **D**, respectively. Cultivation was carried out in 2 L flasks containing 500 mL of medium and incubated at 30 °C. Biomass was measured as dry weight as described in “Materials and methods” section. Taxol and baccatin III were quantified using CIEIA (monoclonal antibody-based competitive inhibition enzyme immunoassay) assay as described in “Materials and methods” section. SigmaPlot was used to plot the data graphs. Each point represents means ± SD from three independent experiments. Symbols are: mycelial dry weight (filled diamond), paclitaxel (filled circle) and baccatin III (open circle).

Liquid cultures of *F. solani* in PDB, M1DB, S7B, FBB and MFBB media were tested for evaluation of their effect on the growth of *F. solani* by cultivation for 28 days where biomass was periodically observed at 7-day intervals under both the static and shaking regimes. Figure 2A–D and Supplementary Fig. S5 show the time course of biomass of *F. solani* under these conditions. We observed discrete growth patterns in five media tested proving differential effect of different nutrient conditions on fungal growth. All the five media showed almost linear growth pattern from day 7 to day 28 except for S7B which showed an apparent lack of growth from day 7 onwards. The production of biomass from these cultures ranged between 2 and 12.1 gL^−1^. Based on these observations, we found that FBB resulted in the highest biomass accumulation of 12.1 gL^−1^ and 9.2 gL^−1^ under static and shaking conditions, respectively (Fig. 2A,B) which was followed by MFBB shaking and static conditions at 8.5 gL^−1^ and 7.7 gL^−1^, respectively (Fig. 2C,D).

### Five evaluated media display differential modulation of Taxol and baccatin III production by *F. solani*

The same five liquid culture media when tested for their effects on the Taxol and baccatin III production by *F. solani* showed discrete production patterns over the experimental period of 28 days at 7-day intervals. The DCM extracts of total cultures were quantified for Taxol and baccatin III contents by CIEIA methods with help of standard curve plots (Supplementary Fig. S4A,B). Our data showed the MFBB as the medium supporting the highest production of Taxol and baccatin III at 136.3 µg and 128.3 µg per liter of culture, respectively, under shaking conditions (Fig. 2C,D). On the other hand, S7B supported the lowest yields of Taxol (6.8 µg L^−1^) and baccatin III (4.9 µg L^−1^) at 7th day under the shaking conditions (Supplementary Fig. S5E). Further, moderate concentrations of taxanes were observed in FBB medium at 30.5 µg L^−1^ and 29.3 µg L^−1^ for baccatin III and Taxol, respectively under shaking conditions at 14th day (Fig. 2B). These findings suggest that among the five media examined for the production of Taxol/baccatin III, the MFBB medium proved best under shaking conditions (Fig. 2C,D). Under the static conditions, FBB yielded the highest baccatin III (127.4 µg L^−1^) productions closely followed by S7B (122.3 µg L^−1^), both after 28 days (Fig. 2A, Supplementary Fig. S5E). In PDB medium, baccatin III yield of 83.4 µg L^−1^ was observed at day 21 under static condition (Supplementary Fig. S5A). In comparison, the Taxol production in static cultures of PDB, M1DB and S7B were all remarkably compromised at below 20 gL^−1^ for all day points (Supplementary Fig. S5A,C,E). The shaking cultures of M1D media too had significant concentrations of the Taxol (107.6 µg L^−1^) after 28 days of cultivation and baccatin III (119.6 µg L^−1^) after 21 days of cultivation (Supplementary Fig. S5D).

### Taxol and baccatin III production by *F. solani* are confirmed through high pressure liquid chromatography (HPLC), liquid chromatography-electrospray ionization mass spectrometry (LC-ESI MS) and nuclear magnetic resonance (NMR) spectroscopy

**Figure 3:**
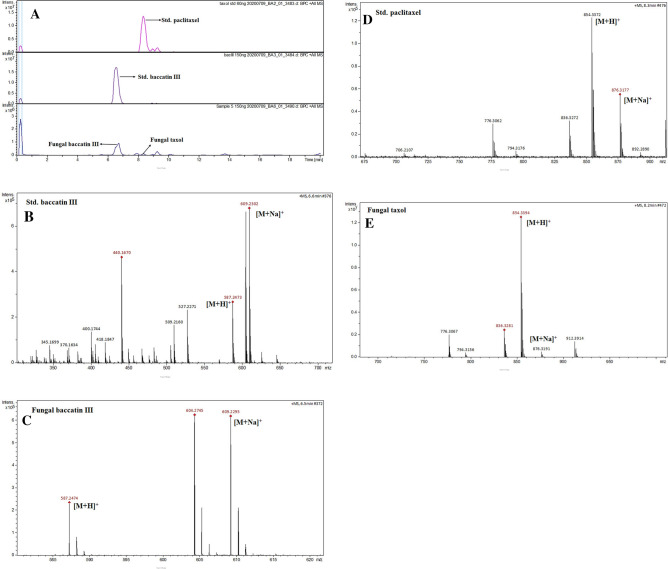
Liquid chromatography (**A**) and mass spectrometry (LC–MS) analysis of standard baccatin III (**B**) and fungal baccatin III (**C**). The mass spectrum of the standard Taxol (**D**) and fungal Taxol (**E**) is shown as well. The samples were processed as described in “Materials and methods” section and the LC peaks at 6.5 and 8.2 min were seen for baccatin III and Taxol, respectively. The diagnostic ion peaks of m/z 587.24 [M+H]^+^ and 609.23 [M+Na]^+^ were observed for baccatin III (**B** and **C**) while the same were observed as m/z 854.33 [M+H]^+^ and 876.31 [M+Na]^+^ for Taxol (**D** and **E**).

**Figure 4:**
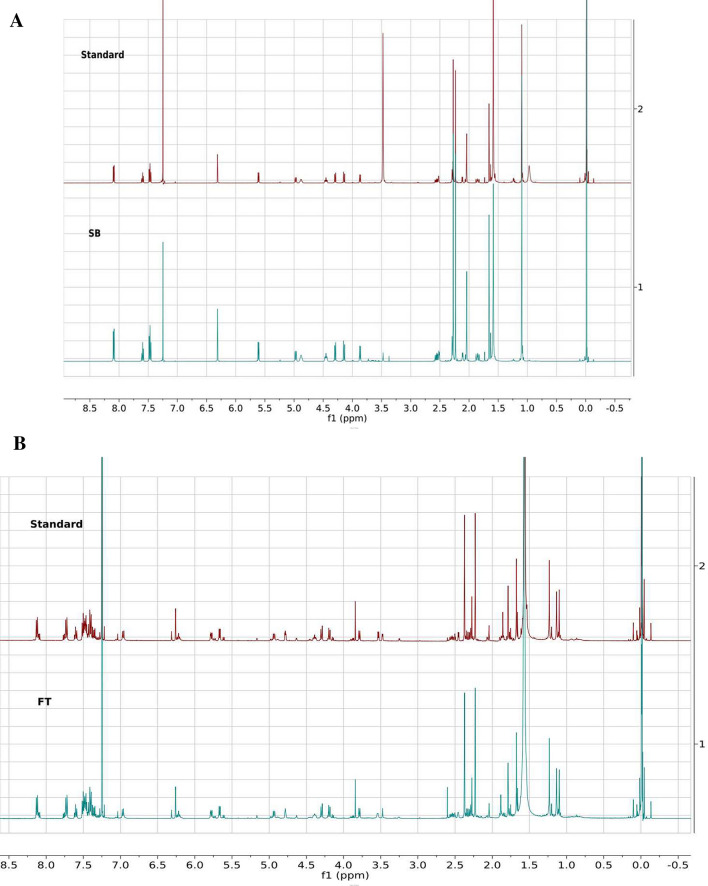
^1^H NMR spectral confirmation of the fungal baccatin III and Taxol. (**A**) NMR spectra of standard and sample (= fungal) baccatin III shown as overlay. (**B**) NMR spectra of standard and fungal Taxol sown as overlay. The NMR spectra overlay files were generated from the raw data files using Mnova software from Mestrelab Research as described in the “Materials and methods”.

We confirmed the Taxol and baccatin III production by *F. solani* in MFBB medium by high pressure liquid chromatography (HPLC), liquid chromatography-electrospray ionization mass spectrometry (LC-ESI MS) as well as nuclear magnetic resonance (NMR) spectroscopy. The HPLC profile of the fungal Taxol and baccatin III with retention times (R_t_) values of 2.27 min and 2.80 min, respectively (Fig. S6A) matched completely with those of authentic standards (Fig. S6B). Figure 3A shows the LC chromatograms for Taxol and baccatin III detection in comparison to standard compounds while the ESI MS spectra of the standard and fungal baccatin III are shown in Fig. 3B and C showing m/z molecular ions of 587.24 [M+H]^+^and 609.22 [M+Na]^+^. The molecular ion peaks of standard and fungal Taxol were seen at m/z 854.33 [M+H]^+^ and 876.31 [M+Na]^+^ (Fig. 3D and E). The ^1^H NMR spectra of the standard compounds and fungal baccatin III and fungal Taxol were similarly found to completely match as shown in Fig. 4 (Fig. 4A,B). Together, these results confirmed the production of Taxol and baccatin III by *F. solani* in the selected medium.

### T13αH and DBAT cDNA fragments of *F. solani* indicate the genetic blueprint of Taxol biosynthetic pathway

**Figure 5:**
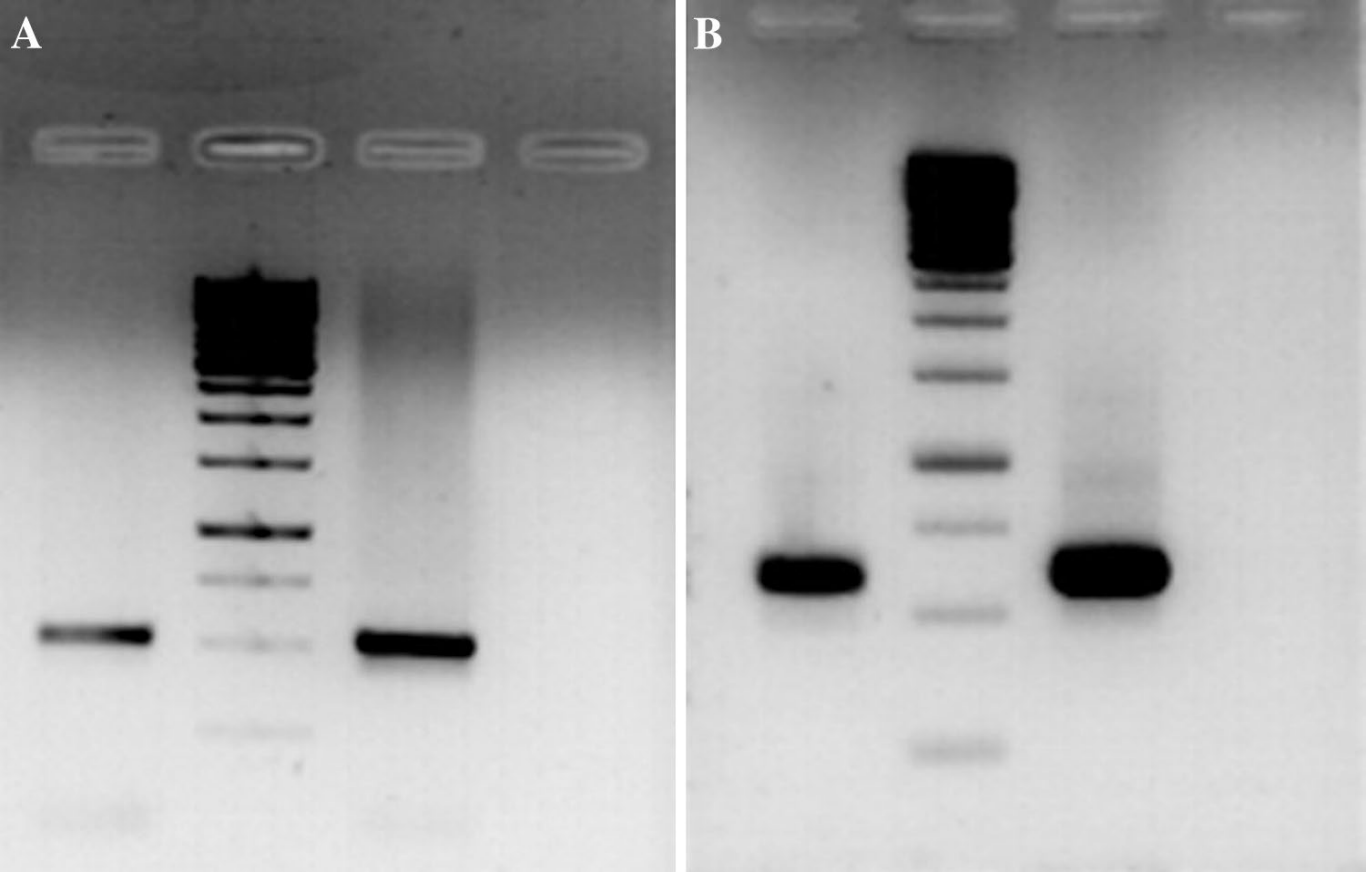
Agarose gel analysis of RT-PCR amplification products of *Fs*T13αH and *Fs*DBAT. The RT reactions were performed using 2 μg of total RNA isolated from 10 days grown *F. solani*. Aliquots of PCR reactions done were analyzed on a 1% agarose gel. The *Fs*T13αH and FsDBAT amplicons of 563 and 621, are shown in (**a**,**b**), respectively. In each case, first, second, third and fourth lanes represent the fungal amplicon, 1 kb DNA marker, the *Taxus celebica* amplicon and the negative control of minus template. The amplicons are seen in between the 500 bp and 750 bp size bands of the marker.

The RT-PCRs with the primers mentioned in the “Materials and methods” section resulted in the amplifications of partial fragments of 563 bp and 621 bp for T13αH (Fig. 5A) and DBAT (Fig. 5B), respectively. The amplified fragments were cloned appropriately and sequenced, and the multiple sequence alignments of the partial fragments showed more than 98% identity at nucleotide level with the respective nucleotide sequences of T13αH *Taxus* × *media* and DBAT of *Taxus cuspidata* (data not shown). These results confirmed the independent genetic signatures of the Taxol biosynthetic pathway in the *F. solani*. The sequences were submitted to GenBank database under the Acc. numbers GU392264 and EF626531 for DBAT and T13H, respectively.

## Discussion

It is an established fact that the growth, sporulation and metabolite production by fungi is influenced by several endogenous cues and environmental factors which ultimately affect the fungal physiology, biochemistry and development. Many of these factors affecting the *F. solani* spore inoculum for biotechnological applications such as nutrition, light and temperature have been an active area of research^22^. The fungal subject of the present study, *F. solani* had been previously reported as a Taxol producing endophytic fungus isolated from *Taxus celebica* from our lab^13^. The present study had been initiated to investigate the role of different media in affecting the spore production and germination characteristics of *F. solani* as well as the potential of these spores as a seed inoculum to assess the Taxol/taxane production by this fungus grown in those media. Our results show a differential effect of five media tested either as semisolids for spore production (PDA, M1DA, S7A, FBA and MFBA) or liquid broths (PDB, M1DB, S7B, FBB and MFBB) for metabolite production on the spore production, their germination and growth characteristics aspects as well as Taxol/taxane production using the produced spores as inoculum by *F. solani* liquid cultures. The maximum radial growth of the *F. solani* was achieved at 30 °C in MFBA medium. Previously, Gupta et al*.*^23^ have reported 28 °C and 34 °C as the incubation temperatures for the highest radial growth of *F. oxysporum* f. sp. *Psidii* as 72.50 mm and 66.5 mm, respectively while the effect of light cycles on the radial growth of *F. oxysporum* f. sp. *Subase* has been reported by Somu and Thammaiah^24^ as 77.4 under alternate 12 h light/dark cycle which declines to 70.8 under continuous light. In comparison, our results show the highest radial growth was observed as 87 mm under alternate 12 h light/dark cycle in MFBA. However, in terms of suitability for spore production, out of the five media tested, PDA supported the maximum spore production followed by M1DA. In line with the results reported by Sharma et al.^25^ reporting the preferential growth and sporulation of *F. oxysporum* f. sp. *lini* in PDA medium under alternate cycles of 12 h light/dark, our results show the same light cycles to induce the highest sporulation by *F. solani*. Similar results have previously been reported by Silva and Teixeira^26–28^.

Germination of spores in liquid media is a vital criterion for the success of fungal cultivation, especially from an industrial standpoint. In this regard, spore inoculum has been reported as a key factor influencing the fermentation stability and yield of final products^29^. For using as a spore inoculum for fermentation to support metabolite production however, the spore viability and morphological characters need to be carefully stdied^30,31^. Our results show that out of the five media tested, M1D medium supported the highest germination of spores as 94.76% and these germinated spores were then used for *F. solani* cultivation in five different production media for Taxol/taxane production.

Bhuyan et al.^32^ have reported an increased penicillin production upon using spores as primary culture inocula. Using the above mentioned M1D germinated spores as inoculum for testing the effect of different media on Taxol and baccatin III production, our results show the highest Taxol production by *F. solani* at 128 μg L^−1^ in MFBB after 21 days of cultivation which is 80 times higher than that of PDB^13^. The same medium supported the highest yield of baccatin III at 136 μg L^−1^. Our data indicates a good correlation between fungal growth and Taxol/baccatin III production and the correspondence to the spore germination was established from these results.

Glucose, usually an excellent carbon source for growth, has been reported to interfere with the biosynthesis of many secondary metabolites^33^. In our study, the higher concentrations of sucrose, NH_4_NO_3_, MgSO_4_, KH_2_PO_4_ and FeCl_3_ in the MFBM medium (Table S1) seems to play an important role in the production of Taxol and baccatin III.

It is apparent that *F. solani* in our study is only a wild strain and thus the yield of Taxol and baccatin III from this fungus could be further enhanced by optimization of the culture media as well as strain improvement including application of genetic engineering. Therefore, in parallel to the spore studies vis-à-vis Taxol/taxane production, we also attempted to clone the Taxol biosynthetic genes from this fungus. Biosynthesis of the Taxol in yew species has been proposed to involve around 20 enzymatic steps from the first committed step of cyclization of geranylgeranyldiphoshate by taxadiene synthase to produce taxadiene^34^ with about eight cytochrome P450-mediated oxygenations and four coenzyme A dependent acylations for building the diterpenoid core^11^. The biosynthesis of Taxol involves approximately nine oxygenation reactions and redundancy in some of these hydroxylase functions has been proposed. The identification and function of several hydroxylases from *Taxus* spp. has been reported including taxoid 2α-, 5α-, 7β-, 10β-, 13α- and 14β-hydroxylases. Five CYP450 oxidoreductases involved in aflatoxin formation in *Aspergillus* have been reported to perform different reactions such as hydroxylation, desaturation or oxidation^35,36^. However, for all the taxoid hydroxylases characterized so far, all have been reported to be mono hydroxylases (Heinig and Jennewein 2009). Interestingly, there are several gaps in our knowledge on the Taxol pathway genes from *Taxus* spp. such as oxidation of hydroxyl-group at C9 or the oxetane ring formation and even more caveats in our understanding of the fungal Taxol pathway, if at all it is identical/similar to that of the *Taxus* plants. Nevertheless, a few of the Taxol biosynthetic pathway genes have been cloned and characterized endophytic fungi^12,37^. As such, we also tried to clone some of these genes from this fungus by using primers designed from plant-based gene sequences. We report partial cloning of two genes from this fungus namely, T13αH and DBAT with the first performing a hydroxylation in the early pathway steps while the later performs an acetylation towards the final pathway steps. A fragment of 563 bp of T13αH hydroxylase characterizing the conversion of 5α-hydroxy-taxadiene into 5α, 13α-dihydroxy-taxadiene^38^ has been cloned. In silico analysis of the partial T13αH cDNA showed a 98% identity with the corresponding gene from *T. cuspidata*. The HXXXD catalytic residues were found to be conserved in *F. solani T13αH* gene. Similarly, the 621 bp of DBAT fragment was found to be 98.5% identical to both the *Taxus* × *media* and *Taxus cuspidata* DBAT genes.

Our results thus demonstrate the potential of the spores produced from the selected media to be used as inoculum for Taxol and baccatin III production by liquid cultures of *F. solani* in addition to confirm the genetic signatures of the Taxol biosynthetic pathway from this endophytic fungus. This significantly adds to our knowledge on the perceived industrial potential of such fungi.

## Materials and methods

### Isolation, maintenance and revival of endophytic *Fusarium solani*

The Taxol producing endophytic fungus *Fusarium solani* used in this study had been earlier isolated from stem cuttings of *Taxus celebica* and characterized by standard microscopic and molecular parameters^13^. Glycerol in water (15% v/v) solution was sterilized by autoclaving at 121 °C for 15 min. A spore suspension was made with sterile 15% glycerol at a concentration of 10^6^ spore per mL in a 2 mL polypropylene cryogenic screw-cap vials containing 0.5 mL of spore suspension. The vials were gradually cooled until frozen in liquid-nitrogen vapor (− 170 °C) and placed at − 70 °C for 4‒5 years^39^. The spores were thawed at 37 °C and plated by adding one drop of the spore suspension in 1 mL broth media on to the PDA petri dishes. The dishes were incubated at 25 °C for 5 days and single colonies were transferred to fresh PDA petri dishes for further experimental use.

### Radial growth and sporulation of *F. solani*

To determine the effects of culture media, temperature and light on mycelial growth as well as sporulation and spore inoculated production of Taxol and baccatin III, the following agar-based semi-solid media were used: PDA (potato dextrose agar), M1DA (Taxol production medium), S7A (antibiotic production medium), FBA (Flask basal agar) and MFBA (Modified flask basal agar). All media compositions are given in Table S1. Semi-solid media were prepared with 2% agar. To test the effects of culture media and temperature on the colony growth, three petri dishes per test medium were inoculated by placing an 8.0 mm diameter agar fungal disc taken from margin of the freshly grown colony of. *F. solani* inversely at the plate center. These were incubated for 12 days at different temperatures 20, 25 30, 35 and 40 °C. The radial mycelial growth was measured at 24 h intervals. The effect of light on mycelial growth was evaluated on the same five solid media throughout the experiments using two Philips ‘Cool white’ (6500 K; 400‒700 wavelength, Philips, India), with energy provided maintained at 50 µE/m^2^/s^40^. Effect of light intensity on the growth of the *F. solani* was studied using 24 h dark, 24 h light, and 12 h dark/12 h light cycles for each tested medium for 12 days at 30 °C with the colony diameters measured at 24 h intervals. Spores were collected from the surface of fungal colonies by gently scraping with a brush and suspended in 10 mL sterile water containing 0.01% (v/v) Tween-20 followed by spore counting with a haemocytometer. These spores would serve as inoculum for later experiments with biomass and Taxol/baccatin III yields in five different liquid media.

### Spore germination assay

Cavity slide culture method was used for studying *F. solani* spore germination^41^. A PDA spore suspension of *F. solani* at a final concentration of 1 × 10^5^ spores mL^−1^ in sterile water were prepared 25 µL of which was mixed with an equal volume of different liquid media (Table S1) in cavity slides separately. The cavity slides were incubated in moist chamber at 28 °C and the spore germination was observed under a light microscope after 24 h. Spores mixed with sterile distilled water served as control. Upon incubation, a drop of lactophenol-cotton blue (40 mL glycerol, 20 mL lactic acid, 20 g phenol and 5 mL of 1% aqueous cotton blue) was added to each slide. The germinated and non-germinated spores were counted under a light microscope and percentage of germination was determined. The morphology of spores and their germination characteristics were observed using phase contrast microscope (Zeiss AX10 Imager A2, Zeiss, Germany) under bright field at × 40 magnification.

### Culture of *F. solani* for production of Taxol and baccatin III

Five different liquid media were investigated for finding the suitable for growth, Taxol and baccatin III production by *F. solani*. Chemical composition of different media is given in Table S1 and is expressed as the amount of individual components added per liter of medium. For the inocula preparation as seed culture, *F. solani* was grown in 25 mL M1D broth by adding (1 × 10^6^) of the spore suspension for 5 days at 25 °C under the shaker condition in dark after which 2% inoculum was transferred into 2 L of each culture medium in 5 L Erlenmeyer flask, separately. The flasks were incubated at 28 °C for 7 to 28 days under static and shaker (150 rpm) conditions.

### Determination of mycelial dry weight

The fungal dry weight was estimated using a pre-weighed filter paper. At the end of the incubation period, the contents of each culture were filtered using Buchner funnel through pre-weighed Whatman No. 3 filter paper and washed with deionized water thrice. The filters were dried overnight at 40 °C in an oven and weighed. The mycelial dry weight at each time point was measured for a minimum of triplicate samples.

### Preparation of *F. solani* culture extracts

For extraction of Taxol and baccatin III, the entire *F. solani* cultures were passed through two layers of cheesecloth to separate the mycelial mass from culture broth and mycelia were dried as described above. The dry mycelial mass was crushed to a fine powder using a mortar and pestle and added back to the culture broth. This combined mixture was extracted with two volumes of dichloromethane. The solvent was removed from the organic residue by rotary evaporation at 35 °C by using rotary vacuum evaporator (Buchi Shimadzu Corporation, Japan). The crude organic extracts thus obtained were dissolved in 1 mL of methanol and filtered through a 0.5 μm filter prior to ELISA (CIEIA) analysis^13^.

### Immunoassay-based quantification of Taxol and baccatin III

Competitive inhibition enzyme immunoassays (CIEIA) were employed for measuring concentrations of Taxol and baccatin III as per the manufacturer’s instructions (Cardax Pharmaceuticals, Hawaii, USA). Here the experiments were performed in triplicates in 96-well microtitre plates. Sensitivity values of 3.5 ng mL^−1^ (Taxol immunoassay kit, Catalog No. TA02) and 10 ng mL^−1^ (baccatin III immunoassay kit, Catalog No. TA03) have been reported by the manufacturer. The organic fungal crude extracts were centrifuged in a microfuge for 10 min to remove any insoluble material. The wells of the 96-well microtitre plates (Laxbro, USA) were suitably coated with either Taxol or baccatin III-protein coating antigen. The plate wells thus coated were blocked with 1% (v/v) BSA and washed with PBS. Solid-phase bound Taxol and baccatin III were incubated with 50 µL of the suitably dissolved samples as well as Taxol and baccatin III standards. These were then challenged with a specific monoclonal antibody provided with each specific immunoassay kit. The Taxol and baccatin III in the samples compete with the respective solid-phase-bound Taxol and baccatin III. These were detected using an alkaline phosphatase-conjugated secondary antibody and p-nitrophenyl phosphate as substrate by detecting the signals at 405 nm measured by a plate reader (Molecular Devices, USA). It was observed that the degree of signal inhibition was proportional to the concentrations of free Taxol and baccatin III present in the samples. Standard curves were plotted for individual assays using the standards supplied with respective kits i.e. Taxol and baccatin III. The values were calculated from these standard curves. Triplicate experiments were performed.

### High-pressure liquid chromatography (HPLC) analysis of standard and fungal baccatin III and Taxol

A Suppelco C18‐column (250 × 4.6 mm × 5 μm) column was used in a Thermo (Germany) HPLC machine with an autosampler and the separation was achieved using an isocratic mobile phase consisting of acetonitrile:water (70:30, v/v). A flow rate of 1 mL min^−1^ was used and the detection of the effluent was monitored at 232 nm. For each sample, 20 μL was injected using the autosampler. Comparison of the retention times with authentic standards of Taxol and baccatin III (Sigma Aldrich., USA) provided detection of the target compounds in the crude extracts.

### Liquid chromatography-electrospray ionization mass spectrometry (LC-ESI MS) and NMR analyses of standard and fungal baccatin III and Taxol

For these experiments, the fungal sample (20 µL) was subjected to liquid chromatography coupled to mass spectroscopy analysis (LC-ESI-QTOF Mass Spectrometer, HPLC System: Dionex Ultimate 3000; Impact HD Bruker Daltonik, GmbH). Samples were run using an isocratic mobile phase of water and acetonitrile (60:40, v/v) on an Agilent poroshell 120 column (4.6 × 150 mm) with SB-C18, 2.7 um particle size at column temperature of 60 °C. The determination mode was in scan mode, the ion–dipole was a positive ion, the ionization mode was electron spray ionization (ESI–MS), end plate offset 500v, capacity 4500v, Nebulizer pressure of 60.0 psi, dry gas 12.0 L min^−1^ and dry temperature 220 °C. The data were acquired over an m/z range of 300–1600 in positive ion mode with a scan rate of 1.0 Hz. The spectra were acquired in auto ESI mode and analysed using the data analysis software Bruker Daltonik (GmbH). The retention time (R_t_) and the molecular mass ion peaks of fungal Taxol and baccatin III were compared with standard Taxol and baccatin III. For the NMR analysis, the purified fungal and standard Taxol and baccatin III were dissolved in deuteriated methanol and NMR spectra were recorded on a Bruker AV III 600 MHz NMR spectrometer at 400 MHz and the overlay files were generated from the raw data files using Mnova software from Mestrelab Research.

### Cloning of segments of Taxol biosynthesis genes taxane 13α-hydroxylase (T13αH) and 10-deacetylbaccatin III-10-O-acetyltransferase (DBAT) from *F. solani*

#### *F. solani* total RNA isolation for cloning of T13αH and DBAT

A seed fungal culture was used to cultivate the fungus in MFBB for 12 days at 30 °C with orbital shaking. Then, fungal mycelia were harvested, the mycelia using the cheese cloth and washing with 50 mM PBS (pH 7.0) and the total RNA was extracted from fungal mycelia. Briefly, 100 mg of the mycelial powder prepared using a chilled mortar/pestle was used per sample as per supplier’s protocol (TRIzol Regent; Invitrogen, California, USA). Any DNA contamination was removed by treating with DNase I (Invitrogen, California, USA). RNase-free water was used for final resuspension. Qualitative and quantitative analyses included agarose gel electrophoresis, spectrophotometer readings of sample absorbance at A260/280 and A260/230 ratios using the Nanodrop 2000 (Thermo Fisher Scientific, California, USA) to assess the purity and quantity of the isolated RNA.

#### cDNA synthesis and cloning of T13αH and DBAT ORF segments

Total RNA isolated from *F. solani* was used as a template for first strand cDNA synthesis in a 20-μL reaction with M-MuLV reverse transcriptase (RT) and oligo (dT)_18_ primer (5′-TTTTTTTTTTTTTTTTTT-3′) as the primer according to the manufacturer’s protocol. The reaction was stopped by heating the reaction mix at 70 °C for 10 min. PCRs were performed using T13αH specific primers T13αHF/T13αHR as 5′-AGATTGTCTCGGCGGCAAAAC-3′/5′-ATAGCTTTCTGGATGGGAGGCCA-3′, respectively and the DBAT specific primers DBAFTF1/DBATR1 as 5′-AGAGATTAAGCCCTCCTCGGA-3′/5′-CCTTCAATCCATGTTGCACGPCR-3′, respectively which had been designed using the conserved regions of the respective gene sequences reported at NCBI database. PCR conditions included an initial denaturation for 2 min followed by 35 cycles of denaturation for 94 °C for 45 s, annealing for 58.9/56 °C for T13αH/DBAT for 30 s and extension for 72 °C for 1 min, and a final extension for 10 min in a total volume of 50 μL containing 75 mM Tris–HCl (pH 8.8), 50 mM KCl, 5 mM MgCl_2_ 2 mM of each dNTP, 20 pmol of each primer, 1 unit of Phusion polymerase (Thermo Fischer) and 2 µL of RT reaction product as a template on an MJ Research Mini Cycler (USA).

### Cloning of PCR amplified partial ORF fragments, sequence confirmation and analysis

PCR products were gel purified and cloned into the pJET1.2 cloning vector (Fermentas) as per the manufacturer’s recommendations by using a vector to insert ratio of 1:3. An aliquot of ligation reaction was used to transform *E coli* DH5α competent cells prepared by the calcium chloride/magnesium chloride method. Transformed colonies were selected on LB medium containing ampicillin (100 µg mL^−1^). The plasmid DNAs were purified using the GenElute Plasmid Miniprep Kit (Sigma-Aldrich, USA) and the insert was sequenced in an automated DNA sequencer (PRISM Ready Reaction DyeDeoxy Terminator Cycle Sequencer; MWG Genomics). For comparison and analysis of the sequence data following programs were used: BLAST^42^, FASTA, GAP, MAP, and SEQED and TRANSLATE of Genetics Computer Group (GCG), Wisconsin, version 7.0^43^. The multiple sequence alignment was performed using the CLUSTALW, European Bioinformatics Institute, at the ExPasy site (http://www.expasy.ch). The Domain analysis for the deduced amino acid sequence was performed using Pfam (Protein families) facility located at the Sanger Centre, UK (http://www.sanger.ac.uk).

### Statistical analysis

All data unless otherwise stated, are presented as mean ± standard deviation (SD) for a minimum of three independent experiments. The statistical significance was analysed using one-way analysis of variance using software GraphPad Prism 5.1 and SigmaPlot at *p*-value < 0.05.

## Supplementary information

Supplementary Information.

## Abbreviations

PDB: Potato dextrose broth
M1DB: Modified 1D broth
S7B: Antibiotic production broth
FBB: Flask basal broth
MFBB: Modified flask basal broth
CIEIA: Competitive inhibition enzyme immunoassays
