# Supplementary material for: Evaluation of spore inoculum and confirmation of pathway genetic blueprint of T13αH and DBAT from a Taxol-producing endophytic fungus
Source: Sci Rep. 2020 Dec 3;10:21139. doi: 10.1038/s41598-020-77605-x (PMC7712836; doi:10.1038/s41598-020-77605-x)
Supplement: Supplementary file 1 — Supplementary Information. [file 41598_2020_77605_MOESM1_ESM.docx]

**Supporting Information**

**Evaluation of spore inoculum and confirmation of pathway genetic blueprint of T13αH and DBAT from a Taxol-producing endophytic fungus**

Balabhadrapatruni V. S. K. Chakravarthi^a#^, Satpal Singh^a#^, Subban Kamalraj^a^, Vijai Kumar Gupta^b^ and Chelliah Jayabaskaran^a^*

#: Equal contribution

a: Department of Biochemistry, Indian Institute of Science, Bangalore-560012, India

b: Biorefining and Advanced Materials Research Center, Scotland's Rural College (SRUC), SRUC Barony Campus, Parkgate, Dumfries DG1 3NE, UK

**Authors' contact information**

1. Balabhadrapatruni V. S. K. Chakravarthi: [chakravarthis@gmail.com](mailto:chakravarthis@gmail.com)
2. Satpal Singh: [satpal82@gmail.com](mailto:satpal82@gmail.com)
3. Kamalraj Subban: [mycolkamal@gmail.com](mailto:mycolkamal@gmail.com)
4. Vijai Kumar Gupta: [vijaifzd@gmail.com](mailto:vijaifzd@gmail.com)
5. Jayabaskaran Chelliah*: [cjb@iisc.ac.in](mailto:cjb@iisc.ac.in)

***Corresponding author:**

Prof. C. Jayabaskaran, Department of Biochemistry,

Indian Institute of Science, Bangalore-560012, India, Tel: +91-80-22932482; Fax: +91-80-23600814; E-mail: [cjb@iisc.ac.in](mailto:cjb@iisc.ac.in)

Table S1. Different media used for the optimization of fungal Taxol and Baccatin III production (This composition of each media with 2% agar served as solid media)

| Medium | pH | Composition (per Liter medium) | Reference |
| --- | --- | --- | --- |
| PDB | 5.1 | Potato infusion – 200 g; D‐Glucose – 20 g | Zhao *et al.*, 2009 |
| M1DB | 5.5 | Ca(NO_3_)2 – 280 mg ; KNO_3_ – 80 mg; KCl – 60 mg; MgSO_4_ – 360 mg; NaH_2_PO_4_ – 20 mg; Sucrose – 30 g; Ammonium tartarate – 5 g; FeCl_3_ – 2 mg; MnSO_4_ – 5 mg; ZnSO_4_ – 2.5 mg; H_3_BO_3_ – 1.4 mg; KI – 0.7 mg; Soytone – 1 g | Strobel *et al.*, 1996 |
| S7B | 6.8 | D‐Glucose – 1 g; D‐Fructose – 3 g; D‐Sucrose – 6 g; Soytone – 1 g; CH_3_COONa – 1 g; Thiamine¬ (B1) – 1 mg; Biotin – 1 mg; Pyridoxal – 1 mg; Ca2+‐ pantothenate – 1 mg; MgSO_4_ – 3.6 mg; Ca(NO_3_)_2_ – 6.5 mg; Cu(NO_3_)_2_ – 1 mg; ZnSO_4_ – 2.5 mg; MnCl_2_ – 5 mg; FeCl_3_ – 2 mg; L-Phenylalanine – 5 mg; C_6_H_5_COONa – 100 mg; 1M KH_2_PO_4_ – 1 mL | Stierle *et al.*, 1996a |
| FBB | - | D‐Glucose – 80 g; NH4NO_3_ – 5 g; MgSO4 – 0.5 mg; KH_2_PO_4_ – 0.5 mg; Cu(NO_3_)_2_ – 1 mg; ZnSO_4_ – 1 mg; FeCl_3_ – 2 mg; CH_3_COONa – 1 g; Thiamine¬ (B1) – 50 mg; L‐tyrosine – 5 mg | Xu *et al.*, 2006 |
| MFBB | - | D‐Glucose – 80 g; NH4NO_3_ – 8 g; MgSO_4_ – 0.7 mg; KH_2_PO_4_ – 0.6 mg; Cu(NO_3_)_2_ – 1 mg; ZnSO_4_ – 1 mg; FeCl_3_ – 5 mg; CH_3_COONa – 2 g; Thiamine¬ (B1) – 50 mg; L‐tyrosine – 5 mg | Present study |

PDB: Potato dextrose broth; M1DB: Modified 1-D broth; S7B: Antibiotic production broth; FBB: Flask basal broth; MFBA: Modified flask basal broth.


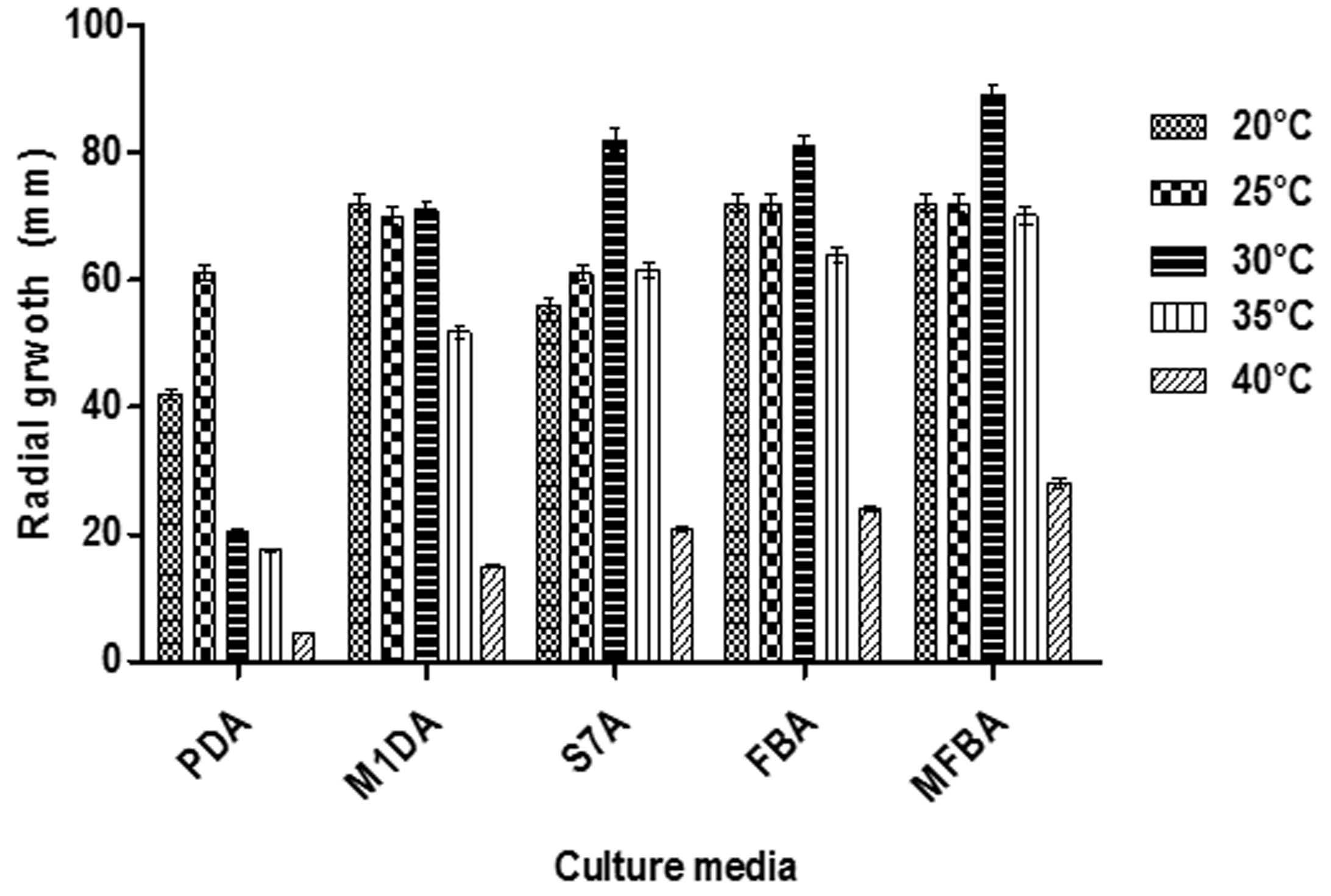


**Figure S1** **Radial growth of *F. solani* at different temperatures on five different solid media after 12^th^ day of incubation**. The fungus was cultured as described in methods. Data are expressed as means ± SD from three in dependent experiments. GraphPad Prism 5.1 was used for data representation. (PDA: Potato dextrose agar; M1DA: Modified 1-D agar; S7A: Antibiotic production agar; FBB: Flask basal agar; MFBA: Modified flask basal agar).


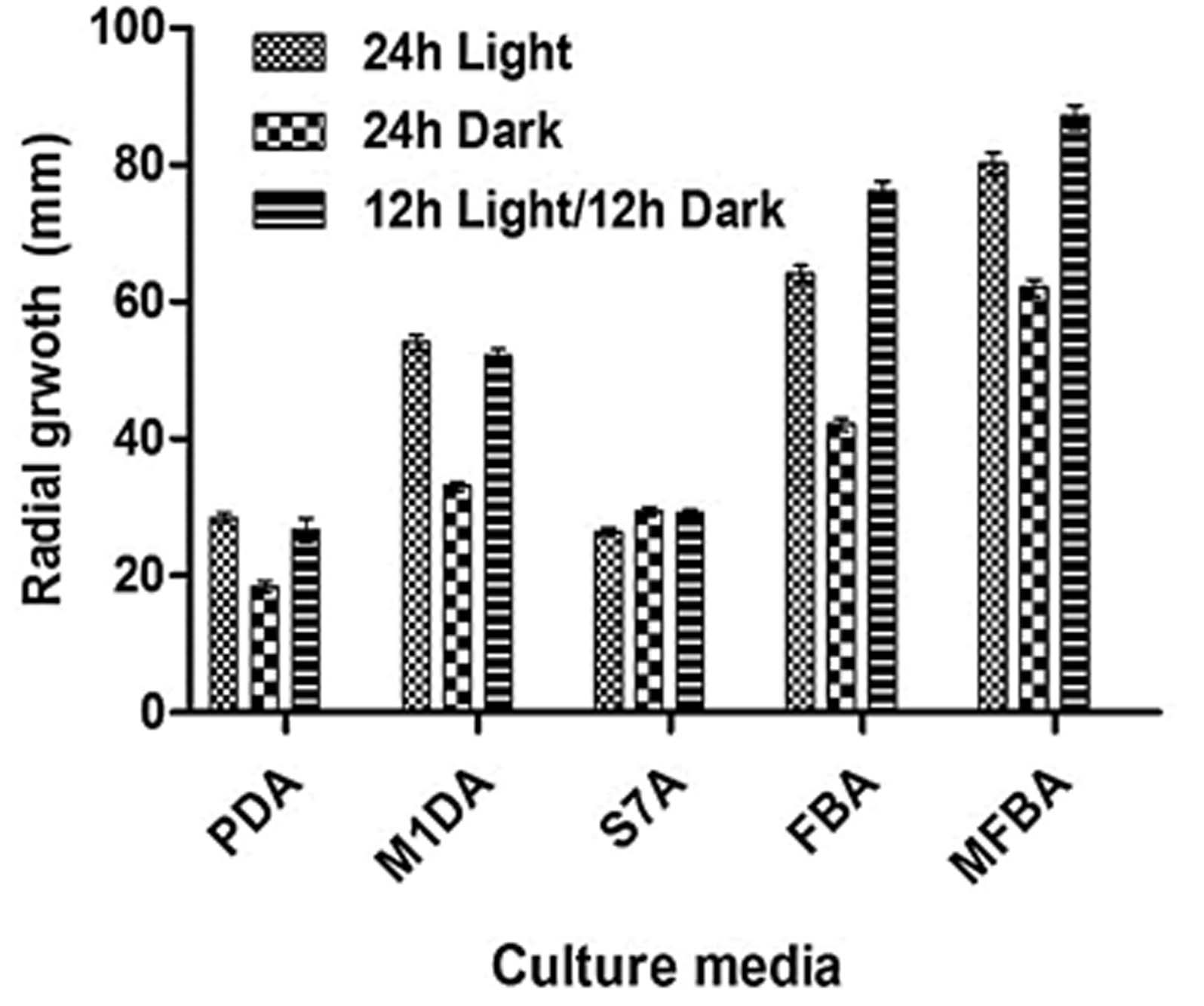


**Figure S2** **Radial growth of *F. solani* on five different solid media at 30°C under three light regimes after 12^th^ day of incubation**. The fungus was cultured as described in methods. Data are expressed as means ± SD from three independent experiments. GraphPad Prism 5.1 was used for data representation. (PDA: Potato dextrose agar; M1-DA: Modified 1-D agar; S7A: Antibiotic production agar; FBA: Flask basal agar; MFBA: Modified flask basal agar).


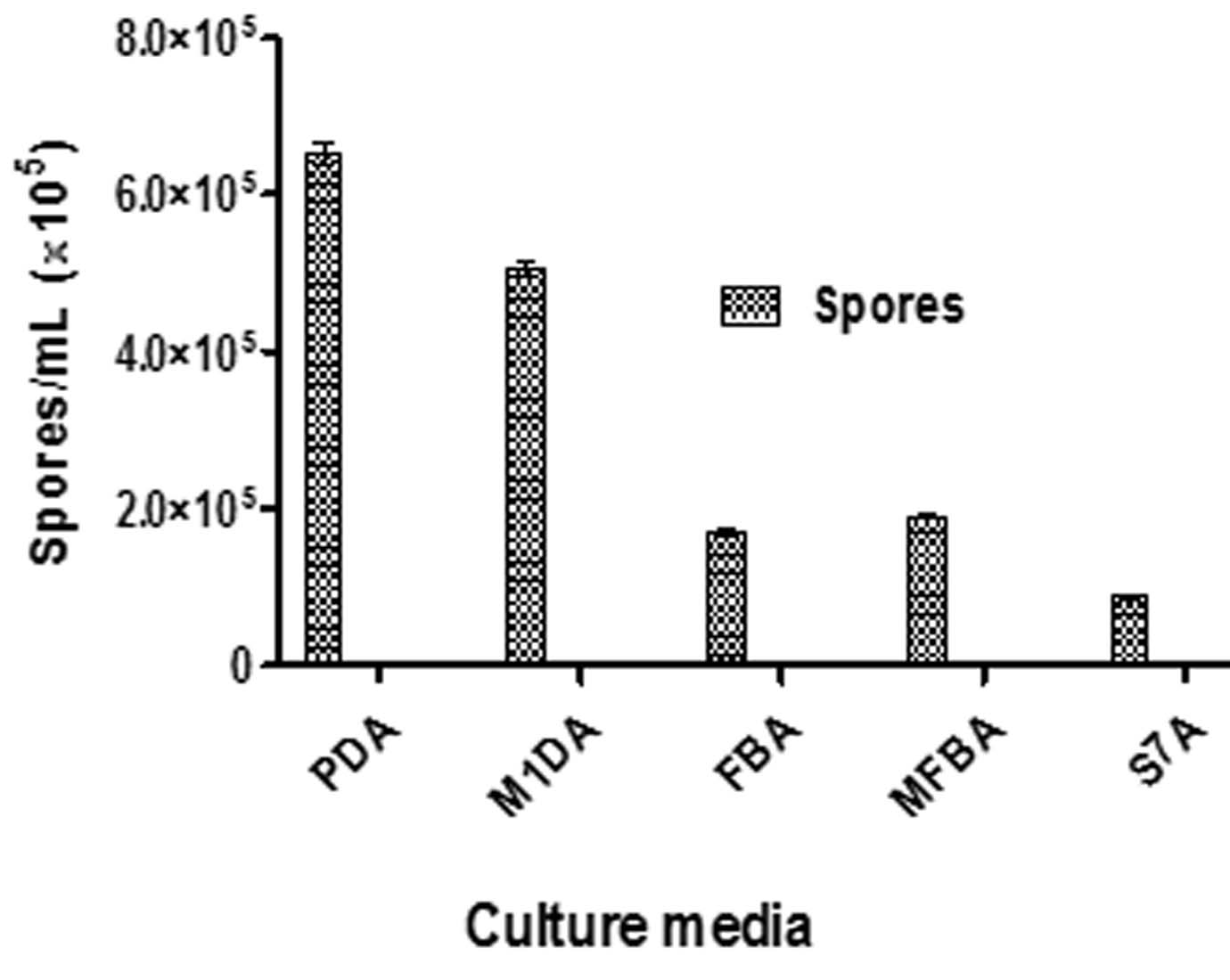


**Figure S3 Sporulation of *F. solani* on different solid media at 30°C under 12h light /12h dark photoperiod after 12^th^ day of incubation**. The fungus was cultured as described in methods. Data are expressed as means ± SD from three independent experiments. GraphPad Prism 5.1 was used for data representation. (PDA: Potato dextrose agar; M1-DA: Modified 1-D agar; S7A: Antibiotic production agar; FBA: Flask basal agar; MFBA: Modified flask basal agar).

**(A)**

**(B)**

**Figure S4 Standard curves for the estimation of Taxol (A), and baccatin III and taxanes (B) by CIEIA ELISA.** Taxol, baccatin III or taxanes solutions were diluted with PBS-T or PBS-T containing 10% MeOH, through the concentration range shown in the graphs, and the samples tested along with controls containing no Taxol, baccatin III or taxanes by indirect CIEIA. Assays were performed as described in Materials and Methods. Inhibition was expressed as % of mean OD_405_ values with and without taxane inhibitor. SigmaPlot was used for data representation. Each point represents an average of triplicate determinations.


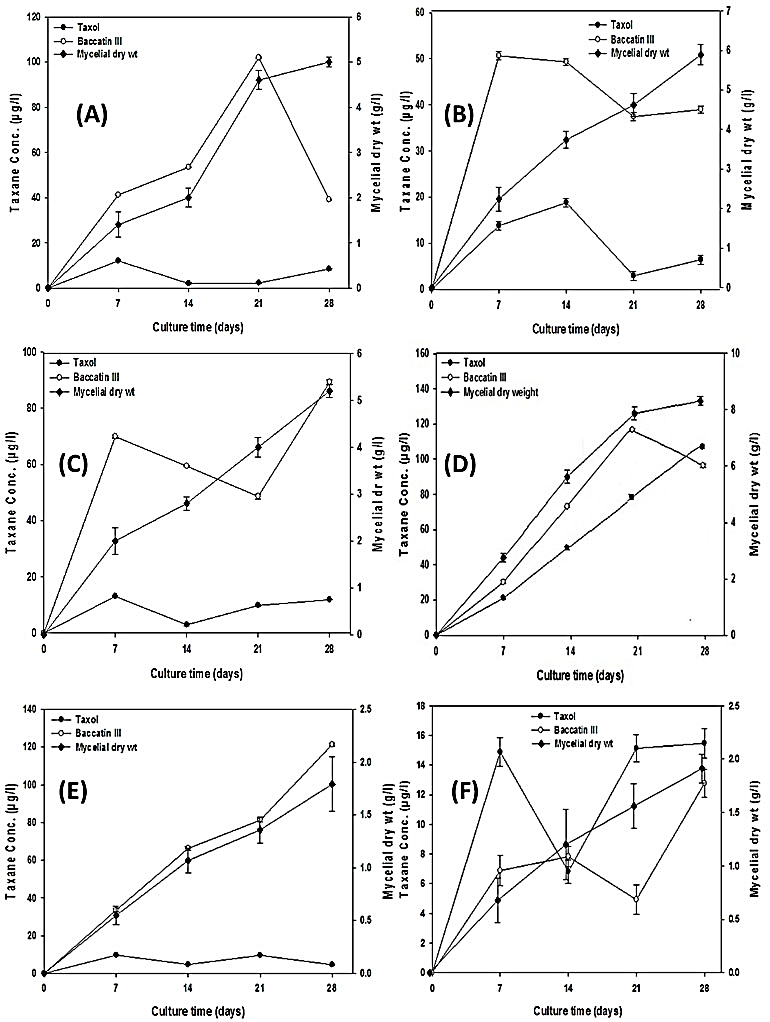


**Figure S5 Time courses of growth, Taxol and Baccatin III production by *F. solani* in PDB under (A) static and (B) shaker conditions**, **M1DB under (C) static and (D) shaker conditions**, **S7B under (E) static and (F) shaker conditions.** Cultivation was carried out in 2 L flasks containing 500 mL of medium and incubated at 30 ^°^C. Biomass was measured as dry weight in samples. Taxol and baccatin III were quantified using CIEIA (monoclonal antibody-based) separately. Each point represents an average of triplicate determinations. Symbols are mycelial dry weight ( ), paclitaxel ( ), baccatin III ( ). SigmaPlot was used for data representation. Each point represents a mean±SD from triplicate experiments.


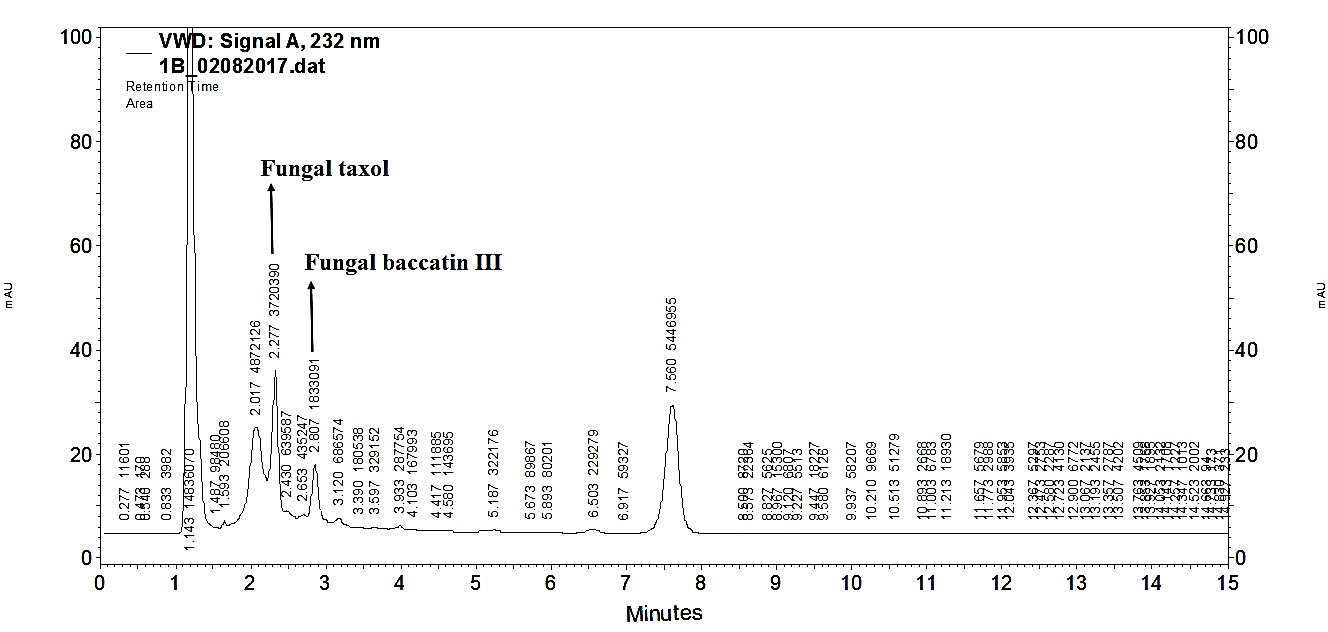


**(A)**


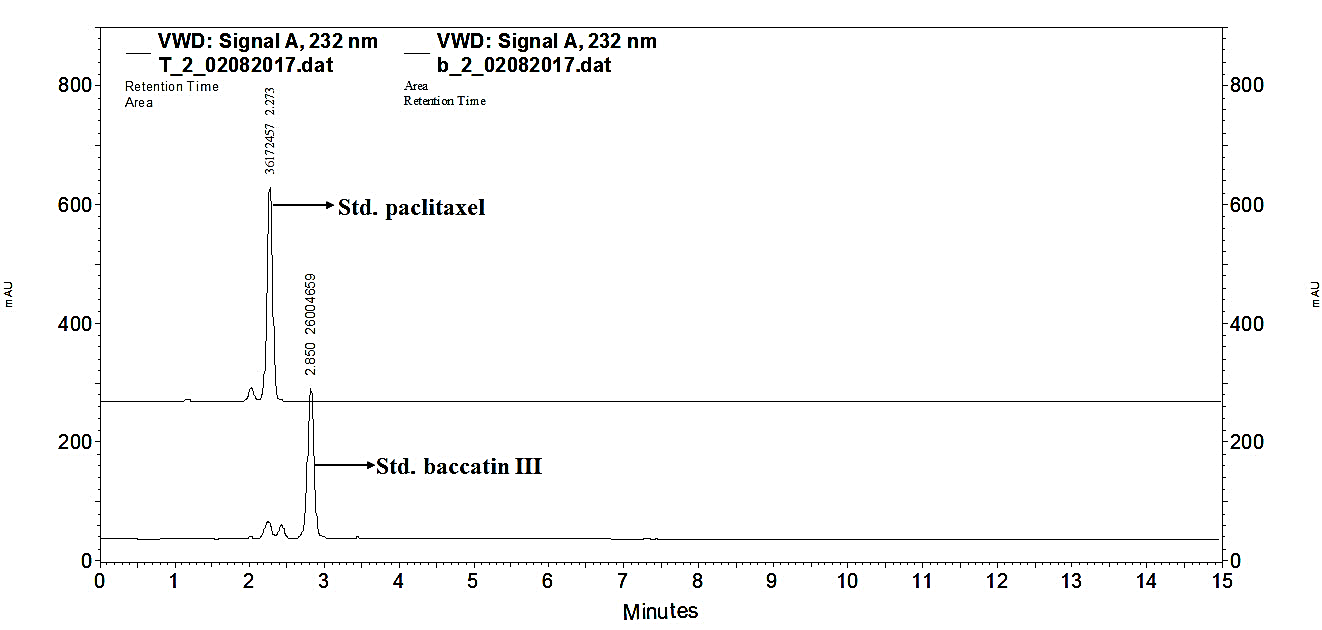


**(B)**

**Figure S6** **HPLC confirmation of baccatin III and Taxol production by F. solani**. (A) HPLC chromatogram of *F. solani* crude culture extracts showing resolved peaks of Taxol and baccatin III at 2.27 and 2.80 minutes, respectively. (B) HPLC chromatograms of the standard Taxol and baccatin III peaks used as references to detect these metabolites in the fungal culture extracts. The details of the experiment are described in the Methods.
